# Eliminating artificial trans fatty acids in Argentina: estimated effects on the burden of coronary heart disease and costs

**DOI:** 10.2471/BLT.14.150516

**Published:** 2015-06-23

**Authors:** Adolfo Rubinstein, Natalia Elorriaga, Osvaldo U Garay, Rosana Poggio, Joaquin Caporale, Maria G Matta, Federico Augustovski, Andres Pichon-Riviere, Dariush Mozaffarian

**Affiliations:** a Institute for Clinical Effectiveness and Health Policy (IECS), Ravignani 2024, Buenos Aires, C1414CPV, Argentina.; b Friedman School of Nutrition Science and Policy, Tufts University, Boston, United States of America.

## Abstract

**Objective:**

To estimate the impact of Argentine policies to reduce trans fatty acids (TFA) on coronary heart disease (CHD), disability-adjusted life years (DALYs) and associated health-care costs.

**Methods:**

We estimated the baseline intake of TFA before 2004 to be 1.5% of total energy intake. We built a policy model including baseline intake of TFA, the oils and fats used to replace artificial TFAs, the clinical effect of reducing artificial TFAs and the costs and DALYs saved due to averted CHD events. To calculate the percentage of reduction of CHD, we calculated CHD risks on a population-based sample before and after implementation. The effect of the policies was modelled in three ways, based on projected changes: (i) in plasma lipid profiles; (ii) in lipid and inflammatory biomarkers; and (iii) the results of prospective cohort studies. We also estimated the present economic value of DALYs and associated health-care costs of coronary heart disease averted.

**Findings:**

We estimated that projected changes in lipid profile would avert 301 deaths, 1066 acute CHD events, 5237 DALYs and 17 million United States dollars (US$) in health-care costs annually. Based on the adverse effects of TFA intake reported in prospective cohort studies, 1517 deaths, 5373 acute CHD events, 26 394 DALYs and US$ 87 million would be averted annually.

**Conclusion:**

Even under the most conservative scenario, reduction of TFA intake had a substantial effect on public health. These findings will help inform decision-makers in Argentina and other countries on the potential public health and economic impact of this policy.

## Résumé

**Objectif:**

Estimer l'impact des politiques argentines de réduction des acides gras trans (AGT) sur les cardiopathies coronariennes (CC), les années de vie corrigées du facteur incapacité (AVCI) et les coûts des soins de santé associés.

**Méthodes:**

Nous sommes partis d'une estimation de l'apport de référence en AGT avant 2004 représentant 1,5% de l'apport énergétique total. Nous avons conçu un modèle pour ces politiques, en intégrant cet apport en AGT de référence, les huiles et graisses utilisées pour remplacer les AGT artificiels, les effets cliniques de la réduction des AGT artificiels, les coûts associés ainsi que les AVCI épargnées du fait des accidents coronariens évités. Pour calculer le pourcentage de réduction des CC, nous avons calculé les risques de CC sur un échantillon en population, avant et après la mise en œuvre de ces politiques. Les effets de ces politiques ont été modélisés de trois manières, en fonction des changements projetés: (i) au niveau des profils lipidiques plasmatiques; (ii) au niveau des biomarqueurs lipidiques et inflammatoires et (iii) en fonction des résultats des études prospectives de cohortes. Nous avons également estimé la valeur économique actuelle des AVCI et du coût des soins de santé associés correspondant aux cardiopathies coronariennes évitées.

**Résultats:**

Selon nos estimations, les changements projetés des profils lipidiques devraient permettre d'éviter 301 décès, 1 066 accidents coronariens aigus, 5 237 AVCI et 17 millions de dollars des États-Unis d'Amérique ($US) de dépenses annuelles en soins de santé. À partir des effets défavorables des apports en AGT indiqués dans les études prospectives de cohortes, ce sont 1 517 décès, 5 373 accidents coronariens aigus, 26 394 AVCI et 87 millions de $US de dépenses qui pourraient être évités chaque année.

**Conclusion:**

Même dans le scénario le plus prudent, la réduction de l'apport en AGT a un effet considérable sur la santé publique. Ces résultats permettront d'informer les décideurs en Argentine et dans d'autres pays sur les impacts potentiels de ce type de politiques sur le plan économique et en termes de santé publique.

## Resumen

**Objetivo:**

Estimar el impacto de las políticas argentinas para la reducción de los ácidos grasos de tipo trans (AGT) en las cardiopatías coronarias, los años de vida ajustados en función de la discapacidad (AVAD) y los costes de la atención sanitaria asociados.

**Métodos:**

Se estimó que la ingesta base de AGT antes de 2004 era de 1,5% de la ingesta de energía total. Se construyó un modelo de política que incluía la ingesta base de AGT, los aceites y grasas utilizados para reemplazar los AGT artificiales, el efecto clínico de reducir los AGT artificiales y el coste y los AVAD salvados debido a los casos de cardiopatías coronarias evitadas. Para calcular el porcentaje de reducción de cardiopatías coronarias, se calcularon los riesgos de cardiopatías coronarias en un modelo basado en la población antes y después de la implementación. El efecto de las políticas fue modelado de tres formas, en base a cambios estimados: (i) perfiles de plasma de lípidos; (ii) marcados biológicos inflamatorios de lípidos; y (iii) los resultados de estudios de cohortes prospectivos. También se estimó el valor económico actual de los AVAD y los costes de atención sanitaria asociados a las cardiopatías coronarias evitadas.

**Resultados:**

Se estimó que los cambios estimados en el perfil de lípidos evitarían 301 muertes, 1.066 casos graves de cardiopatías coronarias, 5.237 AVAD y 17 millones de dólares estadounidenses (USD) en atención sanitaria cada año. Basándose en los efectos adversos del consumo de AGT de los estudios de cohortes prospectivos, se evitarían 1.517 muertes, 5.373 casos graves de cardiopatías coronarias, 26.394 AVAD y 87 millones de USD cada año.

**Conclusión:**

Incluso bajo el escenario más conservador, la reducción del consumo de AGT tuvo un efecto sustancial en la salud pública. Estos resultados ayudarán a informar a los responsables de la toma de decisiones en Argentina y otros países sobre el potencial impacto económico y de salud pública de esta política.

## ملخص

**الغرض:**

تقدير أثر السياسات الأرجنتينية للحد من الأحماض الدهنية المتحولة (TFA) على مرض القلبي الوعائي (CHD)، وسنوات العمر المصححة باحتساب مدد العجز (DALYs)، ونفقات الرعاية الصحية المرتبطة بها.

**الطريقة:**

تشير تقديراتنا إلى أن نسبة المدخول الأساسي من الأحماض الدهنية المتحولة قبل عام 2004 كانت تبلغ 1.5% من إجمالي مدخول الطاقة. لذلك، قمنا بوضع نموذج لسياسة تتضمن المدخول الأساسي من الأحماض الدهنية المتحولة والزيوت والدهون المستخدمة لتحل محل تلك الأحماض الدهنية المتحولة، والأثر الإكلينيكي لتقليل الأحماض الدهنية المتحولة والتكاليف وسنوات العمر المصححة باحتساب مدد العجز التي يتم توفيرها بسبب تحاشي وقائع الإصابة بالأمراض القلبية الوعائية. ولكي يتم حساب النسبة المئوية لتقليل الإصابة بالأمراض القلبية الوعائية، فقد احتسبنا مخاطر تلك الأمراض على عينة تستند إلى شريحة سكانية قبل تنفيذ السياسة وبعدها. وتم وضع نماذج لأثر السياسات باتباع ثلاث طرق استناداً إلى التغييرات المتوقعة: (1) في أنماط دهون البلازما، و(2) في الدهون والمحددات الحيوية الالتهابية (Inflammatory biomarkers، و(3) نتائج الدراسات الأترابية الاستباقية (Prospective cohort studies). كما وضعنا تقديرًا للقيمة الاقتصادية الحالية لسنوات العمر المصححة باحتساب مدد العجز وما يرتبط بها من تكاليف للرعاية الصحية لمرض القلب الوعائي الذي تم تحاشي الإصابة به.

**النتائج:**

تشير تقديراتنا إلى أن التغييرات المتوقعة في نمط الدهون سيؤدي إلى تحاشي 301 حالة وفاة، و1066 واقعة إصابة بحالات حادة من الأمراض القلبية الوعائية، وإنقاذ 5237 سنة عمر مصححة باحتساب مدد العجز، و17 مليون دولار أمريكي سنويًا من نفقات الرعاية الصحية. وبناءً على الآثار الجانبية لمدخول الأحماض الدهنية المتحولة التي وردت تقارير بشأنها في الدراسات الأترابية الاستباقية، فسيتم تجنب 1517 حالة وفاة، و5373 واقعة إصابة بحالات حادة من الأمراض القلبية الوعائية، وإنقاذ 26,394 سنة عمر مصححة باحتساب مدد العجز، و87 مليون دولار أمريكي سنويًا.

**الاستنتاج:**

حتى في ظل السيناريوهات الأكثر تحفظًا، فقد كان للحد من مدخول الأحماض الدهنية المتحولة أثر كبير على الصحة العامة. ومن شأن هذه النتائج أن تفيد في تقديم المعلومات الكافية لصناع القرار بالأرجنتين وغيرها من البلدان بشأن الأثر المحتمل على الصحة العامة والآثار الاقتصادية لهذه السياسية.

## 摘要

**目的:**

旨在评估阿根廷境内关于减少使用反式脂肪酸 (TFA) 的政策对冠心病 (CHD)、残疾调整生命年 (DALY) 和相关医疗保健成本产生的影响。

**方法:**

我们估计出 2004 年之前对反式脂肪酸 (TFA) 的基准摄入量为总能量摄入的 1.5%。 我们构建了政策模型，包括对反式脂肪酸 (TFA) 的基准摄入量、用于代替人工反式脂肪酸 (TFA) 的油脂、减少使用人工反式脂肪酸 (TFA) 的临床效果，以及因预防冠心病 (CHD) 事件而节约的成本和残疾调整生命年 (DALY)。 为了计算冠心病 (CHD) 减少的百分比，我们在实施前后基于研究人群计算了冠心病 (CHD) 风险。 根据预测的变化，以三种方式模拟政策的影响： (i) 血浆中的血脂；(ii) 脂质和炎性标记物；以及 (iii) 前瞻性群组研究的结果。 我们还估计了残疾调整生命年 (DALY) 在当下的经济价值，以及预防冠心病的相关医疗保健成本。

**结果:**

我们估计，血脂变化预计每年将会避免 301 人死亡、1066 例急性冠心病 (CHD) 事件和 5237 个残疾调整生命年 (DALY)，并可节约 1700 万美元 (US$) 的医疗保健成本。 基于前瞻性群组研究中所报告的摄入反式脂肪酸 (TFA) 后产生的不良影响，每年可防止 1517 人死亡、5373 例急性冠心病 (CHD) 事件和 26 394 个残疾调整生命年 (DALY)，并可节约 8700 万美元 (US$)。

**结论:**

即使是在最为保守的情况中，减少摄入反式脂肪酸 (TFA) 也可对公众健康产生重大影响。 这些调查结果将有助于让阿根廷和其他国家的决策制定者了解潜在的公众健康问题和这项政策的经济影响。

## Резюме

**Цель:**

Оценить воздействие применяемых в Аргентине политик по уменьшению количества трансжирных кислот в пище (ТЖК) на развитие ишемической болезни сердца (ИБС), на количество лет жизни, утраченных в результате болезни, и на сопутствующие расходы на здравоохранение.

**Методы:**

По нашим оценкам, до 2004 года 1,5% общего количества пищевых калорий приходилось на ТЖК, и эту величину мы приняли за базовый уровень. Мы разработали модель политики, включающую базовый уровень потребления ТЖК, жиры и масла, которыми планировалось заменить искусственные ТЖК, клинический эффект от снижения уровня ТЖК, а также уровень экономии и количество спасенных лет жизни в результате предотвращения случаев ИБС. Для расчета процентной доли снижения ИБС были рассчитаны риски возникновения ИБС для популяционной выборки до и после реализации вышеуказанной политики. Эффект от применения политик моделировался тремя путями в зависимости от предполагаемых изменений: (i) по профилям липидов в плазме, (ii) по липидам и биомаркерам воспалительного процесса и (iii) по результатам проспективных когортных исследований. Мы также оценили текущее экономическое значение спасенных лет жизни и уменьшения сопутствующих расходов на лечение в случае предотвращения ишемической болезни сердца.

**Результаты:**

По предварительным оценкам, предполагаемые изменения в профиле липидов позволят предотвратить 301 смерть, 1066 острых случаев ИБС, спасти 5237 лет жизни и сэкономить 17 млн долл. США на ежегодных расходах на здравоохранение. Если исходить из неблагоприятных последствий употребления пищевых ТЖК, о которых сообщалось в проспективных когортных исследованиях, то ежегодно можно будет предотвратить 1517 смертей, 5373 острых случая ИБС, спасти 26 394 года жизни и сэкономить 87 млн долл. США на медицинских расходах.

**Вывод:**

Даже при самом неблагоприятном сценарии уменьшение употребления ТЖК в значительной мере повлияет на здоровье населения. Эти результаты помогут информировать ответственных лиц в Аргентине и других странах о потенциальном воздействии такой политики на здоровье населения и национальную экономику.

## Introduction

Artificial trans fatty acids (TFAs) are produced during the industrial processing of vegetable oils. The main source of such TFAs is partially hydrogenated vegetable oils.^1^ Consumption of TFAs alters the plasma lipid profile^2^^,^^3^ in such a way that it increases the risk of coronary heart disease (CHD).^4^ A 2% increase in energy intake from TFAs may increase the risk for a coronary event by up to 23%.^3^ Other potential adverse effects of TFAs include systemic inflammation, endothelial dysfunction, insulin resistance and arrhythmias.^2^

Based on these adverse effects, several countries have implemented policies to reduce industrial TFA consumption,^5^ including nutrition guidelines, awareness programmes, voluntary or mandatory labelling of the TFA content of foods and health warning labels. Voluntary or legislated programmes to encourage industry to reformulate food products without TFAs and support the production of healthy alternatives have led to improvements in some countries.^6^ Mandatory food labelling in Canada^7^ and the United States of America^8^ have led some manufacturers to reduce or eliminate artificial TFAs in their products. However, many food products still contain such TFAs, especially when served in restaurants, schools, cafeterias and coffee shops.^9^

**Fig. 1:**
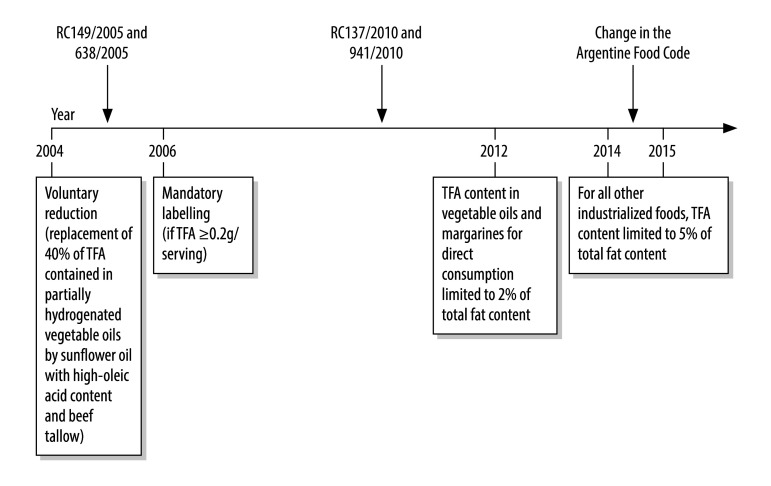
Trans fatty acids regulations in Argentina, 2004–2015

RC: Ministerial Resolution; TFA: trans fatty acids.

In Argentina, before 2004, artificial TFAs were present in most sweet or salty solid snack foods, such as biscuits.^10^ Between 2004 and 2014, Argentina implemented several policies to reduce artificial TFAs. After 2004, the industry voluntarily reformulated foods by replacing approximately 40% of TFAs from partially hydrogenated vegetable oils, mainly with TFA-free sunflower oil with high-oleic acid content.^11^ Regulations enforcing mandatory labelling of artificial TFAs in food were introduced in 2006.^12^ With support from the Pan American Health Organization,^13^^,^^14^ the Argentine Ministry of Health negotiated with industry to eliminate artificial TFAs. The country’s food code was amended,^15^ such that, by the end of 2014, industrially-produced TFAs in food should not exceed 2% of total fats in vegetable oils and margarines and 5% of total fats in other foods (Fig. 1).^16^

Here we estimate the potential reductions in annual CHD events, disability-adjusted life years (DALYs) and associated health-care costs attributable to reductions in artificial TFAs in the diet.

## Methods

The main inputs of the policy model for the analysis were: (i) the estimated baseline intake of TFAs before 2004; (ii) the types of alternative oils and fats used to replace TFAs; (iii) the effects of the improvements in plasma lipid profile on CHD risks and (iv) the health-care costs and DALYs saved due to averted fatal and nonfatal CHD events. Although our study is not a full economic evaluation, we used the CHEERS statement as a guide for reporting.^17^

### Baseline intake of TFAs

To identify estimates of baseline TFA intake in Argentina and the fats used to improve the dietary fat profile between 2004 and 2014, we conducted a literature search using MEDLINE, Embase, LILACS databases and official documents from the government, academia, industry and other public and private organizations. For the database searches, we used the search string “trans fat OR trans fatty acids OR partially hydrogenated oils OR partially hydrogenated fat AND Argentina”.

Because TFAs cannot be replaced on a 1:1 basis with other specific fatty acids, the unit of replacement was partially hydrogenated vegetable oils (comprised of various fatty acids, including TFAs). Thus, we evaluated both the total partially hydrogenated vegetable oils consumed and the usual proportion of TFAs in partially hydrogenated vegetable oils during 2004–2014. Our search was complemented by a consensus meeting of local experts and decision-makers including officials from the Ministry of Health, epidemiologists, nutritionists, cardiologists and food engineers closely involved with the oils’ and fats’ suppliers of TFA replacements. They identified key estimates for the model, including the baseline intake of TFA, the proportion of TFA from ruminants and the replacement fats used by industry. Our central estimate of baseline TFA consumption in 2004 was 1.5% of total energy intake, with a lower limit of 1%^18^ and an upper limit of 3%.^14^ The most common replacement oil was sunflower oil with high-oleic acid content (base case estimate 42.0%; range: 33.6–50.4), followed by interesterified fats (18.0%; range: 14.4–21.6) and beef tallow (12:0% range: 9.6–14.4; Table 1).

**Table 1:** Baseline TFA intake and replacements; epidemiological and cost inputs

Input	Base case (range)	Probability distribution	Source
**TFA intake related**			
TFA intake before 2004, E%	1.5 (1.0 to 3.0)	Normal (mean: base; SD:10% of base)	Consensus panel of experts^14^^,^^18^^,^^19^
Ruminant TFA, %	0.5% of E (0.15 to 0.75)	(Beta; alpha: 2; beta: 3)	Consensus panel of experts^19^
TFA content in PHVO, %	40.0 (30.0 to 50.0)	Minimum extreme (min: 30; max: 50; likeliest: 45; scale: 4.5)	Consensus panel of experts
Replacement by sunflower oil with high-oleic acid content, %	42.0 (33.6 to 50.4)	Normal (min: 0%; max: 100%; mean: 42%; SD: 4%)	Consensus panel of experts
Replacement by beef tallow, %	12.0 (9.6 to 14.4)	Proportionally adjusted to variations of % HOSO	Consensus panel of experts
Replacement by sunflower oil with high-stearic acid content, %	3.5 (2.8 to 4.2)	Proportionally adjusted to variations of % HOSO	Consensus panel of experts
Replacement by sunflower oil and soybean oil, %	3.0 (2.4 to 3.6)	Proportionally adjusted to variations of % HOSO	Consensus panel of experts
Replacement by interesterified fats, %	18.0 (14.4 to 21.6)	Proportionally adjusted to variations of % HOSO	Consensus panel of experts
Replacement by palm oil, %	10.8 (8.6 to 12.9)	Proportionally adjusted to variations of % HOSO	Consensus panel of experts
Replacement by lauric fats, %	10.8 (8.6 to 12.9)	Proportionally adjusted to variations of % HOSO	Consensus panel of experts
**Epidemiological**			
Effects of fats on TC/HDL-C			
Change TFA to SFA	−0.031 (−0.045 to −0.017)	Normal (min: −0.045; max: −0.017; mean: −0.031; SD: 0.007)	Estimates from Mozaffarian and Clarke^2^
Change TFA to MUFA	−0.054 (−0.072 to −0.036)	Normal (min: −0.072; max: −0.036; mean: −0.054; SD: 0.009)	Estimates from Mozaffarian and Clarke^2^
Change TFA to PUFA	−0.067 (−0.085 to −0.049)	Normal (min: −0.085; max: −0.049; mean: −0.067; SD: 0.009)	Estimates from Mozaffarian and Clarke^2^
Change SFA to MUFA	−0.029 (−0.043 to −0.015)	Normal (min: −0.043; max: −0.015; mean: −0.029; SD: 0.007)	Estimates from Mozaffarian and Clarke^2^
Change SFA to PUFA	−0.035 (−0.049 to −0.021)	Normal (min: −0.049; max: −0.021; mean: −0.035; SD: 0.007)	Estimates from Mozaffarian and Clarke^2^
Change MUFA to PUFA	−0.006 (−0.020 to 0.008)	Normal (min: −0.020; max: 0.08; mean: −0.020; SD :0.007)	Estimates from Mozaffarian and Clarke^2^
Effect of TFA replacements on other biomarkers (dietary trials)	2.92 (NA)	Normal (min: 2.33; max: 3.5; mean: 2.92; SD: 0.292)	Estimates from Mozaffarian and Clarke^2^
Effect of TFA replacements from cohort studies	5.04 (NA)	Normal (min: 4.03; max: 6.05; mean: 5.04; SD: 0.50)	Estimates from Mozaffarian and Clarke^2^
Case fatality rate AMI men, %	44.0 (35.2 to 52.8)	Normal (min: 35.2%; max: 52.8%; mean: 44%; SD: 4.4%)	Salomon et al.^20^
Case fatality rate AMI women, %	38.0 (30.4 to 45.6)	Normal (min: 30.4%; max: 45.6%; mean: 38%; SD: 3.8%)	Salomon et al.^20^
Case fatality rate ACS men, %	14.7 (11.7 to 17.6)	Normal (min: 11.7%; max: 17.6%; mean: 14.7%; SD: 1.5%)	Estimated from Bazzino et al.^21^ and Salomon et al.^20^
Case fatality rate ACS women, %	12.7 (10.1 to 15.2)	Normal (min: 10.1%; max: 15.2%; mean: 12.7%; SD: 1.2%)	Estimated from Bazzino et al.^21^ and Salomon et al.^20^
Total AMI deaths (n)	17 942 (NA)	NA	National statistics from MoH
Total CHD deaths (n)	24 875 (NA)	NA	National statistics from MoH
**Cost**			
Cost per AMI event, US$	5 765 (4 612 to 6 918)	Normal (min: 0; mean: 5765.4; SD: 576.5)	Health system costs average^22^^–^^27^
Cost per ACS event, US$	6 416 (5 133 to 7 699)	Normal (min: 0; mean: 6416; SD: 641.6)	Health system costs average^22^^–^^27^
Annual costs per follow-up and treatment, US$	1 199 (959 to 1 439)	Normal (min: 0; mean: 1199; SD: 119.9)	Health system costs average^22^^–^^27^
Programmatic costs, US$	129 001 (NA)	NA	Personal communication (MoH estimates)

ACS: acute coronary syndrome; AMI: acute myocardial infarction; CHD: coronary heart disease; E: energy intake; HOSO: high-oleic sunflower oil; MoH: ministry of health; MUFA: monounsaturated fatty acids; NA: not applicable; PHVO: partially hydrogenated vegetable oils; PUFA: polyunsaturated fatty acids; SD: standard deviation; SFA: saturated fatty acids; TC/HDL-C: total cholesterol/high-density lipoprotein cholesterol; TFA: trans fatty acids; US$: United States dollars.

Notes: Discount rates of 5% (range 0–10) were used.^28^ We used the average conversion rate during 2012, which was US$ 1 to 4.55 Argentine dollars.^29^

### Changes in lipid profile

Improvements in the plasma lipid profile were expected to result in improvements in CHD risks. We assessed the relevant changes in plasma lipid profiles and other biomarkers of CHD risk based on meta-analyses of controlled dietary feeding trials.^2^^,^^4^ These estimates were used to drive projections of CHD risks, as outlined below.

### CHD risk

To estimate reductions in CHD risk in the national population, we adapted a cardiovascular risk calculator, based on the Framingham risk equation and ASSIGN scores.^30^ We used individual level data on CHD risk factors from a national prospective cohort study.^31^ The study collected baseline data in 2011–2012 on age, gender, smoking, systolic blood pressure, diabetes, left ventricular hypertrophy and the ratio total cholesterol (TC)/high-density lipoprotein cholesterol (HDL-C). We combined these results with demographic data for Argentina using the 2010 census to create a national CHD risk profile.^32^

According to the consensus of our expert panel, between 2004 and 2014, most of the partially hydrogenated vegetable oils in the diet were replaced by healthier fats. In 2011–2012, when the prospective cohort study took place, 75% had been replaced. We used the observed TC/HDL-C ratio for each person in the 2011–2012 cohort study to calculate the expected TC/HDL-C ratios in 2004 and 2014. This calculation was based on the estimated baseline intake of TFA, the established effects of TFA on the TC/HDL-C ratio and the types and percentages of different fats/oils used by industry for replacements. According to the distributions of TC/HDL-C and other risk factors in the population in 2004 and 2014, we calculated the difference in the CHD risk between both years. 

Three alternative scenarios were analysed: (i) the effects of improved dietary fat profile on the ratio of TC/HDL-C and the relation of this ratio to the incidence of CHD (scenario 1);^33^ (ii) the CHD risk reduction through changes in other biomarkers such as apolipoprotein (apo) B, ApoA1, lipoprotein (a), triglycerides and C-reactive protein (scenario 2); and (iii) the reported relation of TFA intake, substituted for carbohydrate intake, with the incidence of CHD in a pooled analysis of prospective studies and attributed to several pathophysiological effects of TFA (scenario 3).^2^

### Mortality from CHD

We estimated the annual number of deaths caused by CHD using national mortality statistics for 2010. We included deaths coded according to the International Classification of Diseases (ICD-10) as I20–I25. We also assumed that 80% of the sudden deaths (ICD-10 code R96) were due to CHD.^34^^,^^35^ We increased the number of CHD deaths by 21.5%, to account for the underreporting of CHD as a cause of death.^36^

The difference in CHD risk predicted by the cardiovascular risk calculator was calibrated to the annual mortality from CHD. We assumed that the reduction in CHD deaths was proportional to the difference in estimated CHD risk. We also assumed that the difference in 10 year-CHD risk was equally distributed in each year of the decade 2004–2014, by age and sex.

### Morbidity from CHD

Total acute CHD events – fatal and non-fatal acute myocardial infarctions and acute coronary syndrome – were estimated from national data on CHD deaths based upon sex-specific 28 day-CHD case-fatality rate for acute myocardial infarctions in southern Latin America (38% in women; 44% in men).^36^ For acute coronary syndrome, we used one-third of the case-fatality rate of acute myocardial infarctions, according to local sources.^21^ All values were calibrated by age-sex hospital case-fatality rate in Argentina, obtained from the national hospital discharge registry for the public sector.^37^

### Calculation of DALYs

We calculated DALY using individual equations for years of life lost (YLL) and years of life with disability (YLD) according to the Global Burden of Disease Study.^38^ Briefly, YLL were calculated from national health statistics as the difference between local life expectancy and age at death. YLD is the product of disability weight and length of survival with disability for CHD events. Disability weights for acute myocardial infarctions and acute coronary syndrome were considered equal.^20^ Survival length was estimated using the software DISMOD II (World Health Organization (WHO), Geneva, Switzerland).^39^ Finally, DALYs were reported with discounting at a 5% rate.

### Costs

Cost inputs for the model were costs of acute CHD events, their follow-up and programmatic costs. A micro-costing approach was undertaken considering a health system perspective. Identification of resources related to CHD events, quantities and utilization rates were obtained from secondary local sources^22^^–^^27^ and unit costs were derived from public, social security, and private tariffs of local health insurance institutions.

Costs of annual management of non-fatal CHD were calculated from the individual's age at the episode to the average Argentine life expectancy, by age and gender, and discounted at a 5% annual rate.^28^ Finally, costs borne by the Ministry of Health for the implementation of annual surveillance and monitoring of the compliance of the industry with the regulations were also estimated, and included costs of personnel, food analysis, and onsite training at food companies (Daniel Ferrante, Ministry of Health, personal communication, 2013).

All costs were converted to United States dollars, corresponding to the exchange rate of 2012.^29^

### Sensitivity analyses

**Fig. 2:**
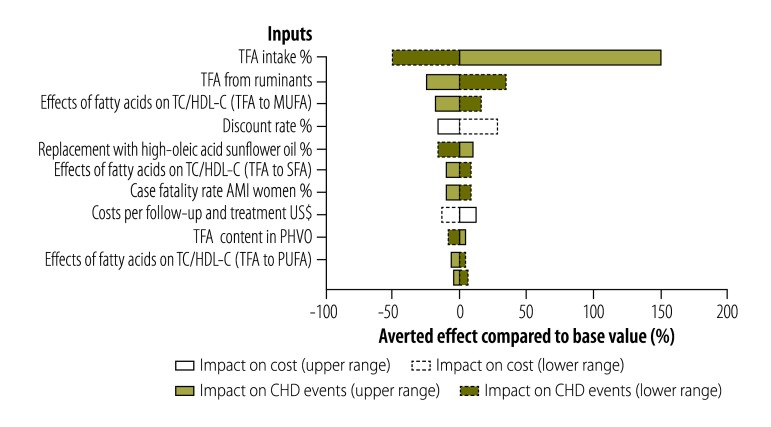
Deterministic sensitivity analysis of the parameters used to estimate the impact of trans fatty acids’ regulations in Argentina, 2004–2014

AMI: acute myocardial infarction; CHD: coronary heart disease; MUFA: monounsaturated fatty acids; PHVO: partially hydrogenated vegetable oils; PUFA: polyunsaturated fatty acids; SFA: saturated fatty acids; TC/HDL-C: total cholesterol/high-density lipoprotein cholesterol; TFA: trans fatty acids; US$: United States dollars.

To evaluate parameter uncertainty, we performed sensitivity analyses according to established guidelines.^40^ A deterministic sensitivity analysis was first performed to evaluate the uncertainty related to specific parameters and their relative importance, depicted in a tornado analysis (Fig. 2). Ranges used for the parameters were extracted from the published literature or expert opinions. To assess global uncertainty, a probabilistic sensitivity analysis was performed, incorporating the main parameters and their distributions. Uncertainty in results was reported using 95% confidence intervals (CI) based on 1000 Monte Carlo simulations. All model inputs including TFA-related, epidemiological and costs parameters are shown in Table 1.

## Results

Mortality, case-fatality and acute coronary events per 100 000 population are shown in Table 2. Based on an estimated 24 875 deaths from CHD in 2010, we estimated 83 830 CHD acute events in Argentina in people older than 34 years old. The results reported here assume a baseline consumption of 1.5% of total energy intake as TFA in 2004.

**Table 2:** Cardiovascular disease events, Argentina, 2010

Event	No. of persons at risk^a^	No. of events	Incidence per 100 000 population	No. of deaths^b^	Mortality per 100 000 population	Case-fatality rate,^c^ %
**Men**						
AMI	–	23 669	302.713	10 414	133	44.0
Sudden death	–	867	11.09	867	11	100.0
ACS	–	21 649	276.87	3 140	40	14.5
Total	7 818 921	46 185	590.681	14 421	184	31.2
**Women**						
AMI	–	19 809	220.08	7 527	84	38.0
Sudden death	–	652	7.25	652	7	100.0
ACS	–	17 184	190.91	2 274	25	13.2
Total	9 000 933	37 645	418.24	10 453	116	27.8
**All**						
AMI	–	43 478	258.49	17 941	107	41.3
Sudden death	–	1 519	9.04	1 520	9	100.0
ACS	–	38 833	2687.53	5 414	32	12.0
**Total**	**16** **819** **854**	**83** **830**	**498.40**	**24** **875**	**148**	**29.7**

AMI: acute myocardial infarction; ACS: acute coronary syndrome.

^a^ Based on 2010 national census.

^b^ Reported by Ministry of Health 2010.

^C^ Average case-fatality rate of an age-calibrated function based on Salomon et al.^20^ and Bazzino et al.^21^

Note: Population older than 34 years.

Based on the most conservative scenario of TFA replacements only influencing CHD events through changes in the TC/HDL-C ratio (scenario 1), we estimated 301 CHD deaths, 572 acute myocardial infarctions, 1066 acute CHD events and 5237 DALYs averted after 2014, compared with the expected events if the policy had not been implemented (Table 3). In addition, more than US$ 17 million would be saved annually due to averted acute CHD events and lower costs of chronic treatment and follow-up.

**Table 3:** Annual CHD deaths and CHD acute events and DALYs averted, and costs savings attributable to the full implementation of the policy

Scenario	No. of CHD deaths averted (95% CI)	No of AMI deaths averted (95% CI)	No of acute CHD events averted (95% CI)	Reduction of CHD events, % (95% CI)	No. of DALYs averted (95% CI)	Total costs saved, million US$ (95% CI)
**Scenario 1: Based only on the effect of TFA replacements on the ratio of TC/HDL-C**						
Base case – 1.5% baseline TFA intake	301 (233 to 433)	572 (443 to 823)	1 066 (875 to 1 623)	1.26 (1.03 to 1.92)	5 237 (4 461 to 8 282)	17.3 (14.5 to 28.7)
Lower limit 1.0%	151 (109 to 273)	286 (207 to 519)	533 (408 to 1 023)	0.63 (0.48 to 1.21)	2 619 (2 081 to 5 220)	8.6 (6.7 to 17.9)
Upper limit 3.0%	752 (571 to 937)	1 429 (1 086 to 1 781)	2 663 (2 142 to 3 515)	3.15 (2.53 to 4.15)	13 087 (10 929 to 17 941)	43.2 (35.0 to 62.4)
**Scenario 2: Scenario 1 plus the effects of TFA replacements on other CHD biomarkers in controlled trials**						
Base case – 1.5% baseline TFA intake	878 (652 to 1 328)	1 668 (1 238 to 2 523)	3 109 (2 442 to 4 978)	3.67 (2.89 to 5.88)	15 271(12 459 to 25 395)	50.5 (40.5 to 87.1)
Lower limit 1.0%	439 (307 to 822)	835 (584 to 1 563)	1 555 (1 190 to 2 984)	1.84 (1.41 to 3.53)	7 637 (5 871 to 15 725)	25.2 (19.7 to 52.2)
Upper limit 3.0%	2 192 (1 577 to 2 871)	4 167 (2 997 to 5 458)	7 764 (6 245 to 10 249)	9.17 (7.38 to 12.11)	38 163 (30 165 to 54 987)	126. 2 (102.2 to 182.1)
**Scenario 3: Based on the observed relationship of TFA replacements with clinical CHD events in prospective cohort studies**						
Base case - 1,5% baseline TFA intake	1 517 (1 118 to 2 285)	2 884 (2 124 to 4 343)	5 373 (4 191 to 8 568)	6.35 (4.95 to 10.12)	26 394 (21 376 to 43 713)	87.3 (69.1 to 150.8)
Lower limit 1.0%	759 (525 to 1 427)	1 442 (997 to 2 712)	2 687 (2 056 to 5 158)	3.18 (2.43 to 6.09)	13 199 (10 031 to 27 294)	43.67 (34.0 to 90.2)
Upper limit 3.0%	3 788 (2 708 to 4 944)	7 202 (5 148 to 9 399)	13 419 (10 794 to 17 713)	15.86 (12.76 to 20.93)	65 958 (51 835 to 94 697)	218.1 (176.6 to 314.7)

AMI: acute myocardial infarction; CHD: coronary heart disease; CI: confidence interval; DALY: disability-adjusted life-years; TC/HDL-C: total cholesterol/high-density lipoprotein cholesterol; TFA: trans fatty acids; US$: United States dollars.

Notes: We used the average conversion rate during 2012, which was US$ 1 to 4.55 Argentine dollars.^29^ Biomarkers included apolipoproteins, triglycerides, lipoprotein (a) and C-reactive protein.

When effects of TFA on CHD were calculated considering additional effects on other biomarkers (scenario 2), under the central estimate of 1.5% energy intake of TFA, a total of 3109 acute CHD events, 15 271 DALYs, and more than US$ 50 million in costs will be averted after 2014. If the effects of TFA on CHD were based on observed relationships with clinical events reported in prospective cohort studies (scenario 3), which may more fully account for the various effects of TFA, 1517 CHD deaths, 2884 acute myocardial infarctions, 5373 acute CHD events and 26 394 DALYs were averted, resulting in estimated savings of USD 87 million (Table 3). The proportion of events averted by the artificial TFA reduction policy in 2014 ranged from 1.26% (scenario 1) to 6.35% (scenario 3) of total CDH events (Table 3). The estimated reductions in CHD were sensitive to the assumed baseline TFA intake in 2004 (Fig. 2).

## Discussion

Given the estimated 84 000 annual CHD events in Argentina, at an annual incidence rate of almost 500 cases per 100 000 adults older than 34 years old, the current policy of near elimination of industrial TFA might avert between 1.3% and 6.35% of CHD events each year. The decrease would save between US$ 17 million and US$ 87 million in management of CHD complications and follow-up. Even in the most conservative scenario, the reduction of TFA intake has a substantial public health impact.

Although there is limited information about the distribution of TFA intakes in subpopulations in most countries, it is likely that many subgroups, particularly low-income populations, could have mean TFA intakes considerably higher than the population mean.^41^ There might be subpopulations that consume more industrially processed foods and fast foods with high-TFA content. Legislative strategies to ban artificial TFAs from foods have been more successful than labelling or education as shown in Austria, Denmark, Iceland, Sweden, Switzerland and USA.^9^^,^^41^^–^^43^ In Denmark, the ban on artificial TFAs is thought to have played some part in the decrease of CHD.^11^

WHO has identified removal of artificial TFAs from the food supply as an intervention with favourable return of invested money to reduce the economic impact of noncommunicable diseases in low- and middle-income countries.^44^ However, most such countries have not yet included the restriction of TFAs’ intake as a policy. Governments have been concerned about the feasibility, achievability and public health effect of removing them from the food supply. Thus, little is known about the potential effects on the reduction of CHD burden and cost savings that could be attributable to the implementation of TFA-reduction policies in these countries. Some middle-income countries such as Brazil,^5^ Costa Rica,^5^ India^45^ and Mexico^5^ are following the Argentine example and are introducing policy and surveillance systems to monitor the content of TFA in foods.

A study modelling a legislative intervention to reduce artificial TFA to 0.5% of total energy intake in the United Kingdom of Great Britain and Northern Ireland, estimated that approximately 2700 deaths annually would be prevented, saving the equivalent of approximately 235 million pounds sterling a year.^46^ Another modelling study estimated a similar potential impact of this policy in Ireland.^47^ Unlike these studies, our model is based on individual data on CHD risk from an Argentine population-based sample, calibrated with national statistics, as well as with local data on dietary fat profiles. Moreover, our study is modelling the impact of a policy that is being implemented.

Potential limitations of this study should be considered. First, to calculate CHD risk in Argentina we used a cardiovascular risk calculator.^30^ The calculator is based on equations developed a couple of decades ago when the CHD incidence was higher. This could overestimate absolute risk in light of secular trends towards lower CHD risk.^48^ On the other hand, these risk equations are widely validated for predicting CHD risk. Overestimation would not likely influence our estimates of proportional risk reduction, since relative risks were calibrated with Argentine absolute risks. Second, we used the global percentage estimates to adjust for underreporting of mortality from CHD. Third, costs of food reformulation by industry were not considered, based on our health system perspective. Yet, potential incremental costs for industry to reduce artificial TFA may be at least partly offset by higher pricing or sales due to marketing advantages.^11^ In the USA, switching to newer frying oils that were free of TFA was cost neutral.^38^ Fourth, we did not have precise data on baseline TFA, the level of which would influence results. Conversely, our nutritional inputs, particularly those related to the TFA baseline intake before 2004, and the partially hydrogenated vegetable oils’ replacements used by the industry thereafter, were obtained after a thorough literature search for sources of TFA in Argentina. This information was reviewed by experts to reach consensus on information gaps to derive a reasonable central estimate and appropriate upper and lower bounds.

In conclusion, our findings suggest that artificial TFA reduction interventions, as an example of a nutritional policy aimed to reach the overall population, have beneficial impact on the total burden of CHD in Argentina. These findings will help inform decision-makers in both Argentina and other countries on the potential public health and economic impact of this policy.
